# Supplementary material for: Bigger and Better? Representativeness of the Influenza A Surveillance Using One Consolidated Clinical Microbiology Laboratory Data Set as Compared to the Belgian Sentinel Network of Laboratories
Source: Front Public Health. 2019 Jun 18;7:150. doi: 10.3389/fpubh.2019.00150 (PMC6591264; doi:10.3389/fpubh.2019.00150)
Supplement: Supplementary file 1 [file Data_Sheet_1.pdf]

**Supplemental digital content 1:** Epiflu Isolate IDs and DNA Accession Numbers for the Influenza A (H1N1) Hemagglutinin sequences used in the phylogenetic analyses in this study. Additional to the 39 samples from LHUB-ULB, the following sequences were used: The vaccine strain for the 2015/2016 season (A/California/7/2009 (H1N1)pdm09); all 17 Influenza A (H1N1) sequences identified with Belgium as place of isolation for the same time period and a random selection of an equal number of 55 Influenza A (H1N1) sequences with the UK as place of isolation for the same time period.

| Isolate_Id | DNA Accession no. | |
|---|---|---|
| EPI_ISL_29577 | EPI176504 | Vaccine 2015/2016 |
| EPI_ISL_217490 | EPI737453 | Belgium samples |
| EPI_ISL_217489 | EPI737448 | |
| EPI_ISL_217487 | EPI737446 | |
| EPI_ISL_217486 | EPI737442 | |
| EPI_ISL_215825 | EPI731164 | |
| EPI_ISL_215824 | EPI731163 | |
| EPI_ISL_215823 | EPI731162 | |
| EPI_ISL_215822 | EPI731161 | |
| EPI_ISL_215821 | EPI731160 | |
| EPI_ISL_215820 | EPI731159 | |
| EPI_ISL_215819 | EPI731158 | |
| EPI_ISL_215818 | EPI731157 | |
| EPI_ISL_215817 | EPI731156 | |
| EPI_ISL_213844 | EPI718571 | |
| EPI_ISL_213829 | EPI718483 | |
| EPI_ISL_213827 | EPI718460 | |
| EPI_ISL_213818 | EPI718401 | |
| EPI_ISL_212266 | EPI712137 | United Kingdom samples |
| EPI_ISL_212268 | EPI712150 | |
| EPI_ISL_214435 | EPI722265 | |
| EPI_ISL_214436 | EPI722273 | |
| EPI_ISL_214437 | EPI722281 | |
| EPI_ISL_214438 | EPI722289 | |
| EPI_ISL_214439 | EPI722297 | |
| EPI_ISL_214440 | EPI722305 | |
| EPI_ISL_214441 | EPI722313 | |
| EPI_ISL_214442 | EPI722321 | |
| EPI_ISL_214443 | EPI722325 | |
| EPI_ISL_214444 | EPI722333 | |
| EPI_ISL_214445 | EPI722337 | |
| EPI_ISL_214446 | EPI722341 | |
| EPI_ISL_214447 | EPI722349 | |
| EPI_ISL_214448 | EPI722357 | |
| EPI_ISL_241127 | EPI876576 | |
| EPI_ISL_241128 | EPI876584 | |
| EPI_ISL_241129 | EPI876592 | |
| EPI_ISL_241130 | EPI876600 | |
| EPI_ISL_241131 | EPI876608 | |
| EPI_ISL_241132 | EPI876616 | |
| EPI_ISL_241133 | EPI876624 | |
| EPI_ISL_241134 | EPI876632 | |
| EPI_ISL_241136 | EPI876648 | |
| EPI_ISL_241137 | EPI876656 | |

| | |
|---|---|
| EPI_ISL_241138 | EPI876664 |
| EPI_ISL_241139 | EPI876672 |
| EPI_ISL_241140 | EPI876680 |
| EPI_ISL_241141 | EPI876688 |
| EPI_ISL_241142 | EPI876696 |
| EPI_ISL_241143 | EPI876704 |
| EPI_ISL_241144 | EPI876712 |
| EPI_ISL_241146 | EPI876726 |
| EPI_ISL_241147 | EPI876734 |
| EPI_ISL_241148 | EPI876742 |
| EPI_ISL_241149 | EPI876750 |
| EPI_ISL_241150 | EPI876758 |
| EPI_ISL_241151 | EPI876766 |
| EPI_ISL_241152 | EPI876774 |
| EPI_ISL_241153 | EPI876782 |
| EPI_ISL_241154 | EPI876790 |
| EPI_ISL_241155 | EPI876798 |
| EPI_ISL_241156 | EPI876806 |
| EPI_ISL_241157 | EPI876814 |
| EPI_ISL_241158 | EPI876822 |
| EPI_ISL_241159 | EPI876830 |
| EPI_ISL_241160 | EPI876838 |
| EPI_ISL_241162 | EPI876854 |
| EPI_ISL_241163 | EPI876862 |
| EPI_ISL_241164 | EPI876870 |
| EPI_ISL_241165 | EPI876878 |
| EPI_ISL_241166 | EPI876886 |
| EPI_ISL_241167 | EPI876894 |
| EPI_ISL_241168 | EPI876902 |
