# Supplementary material for: Bigger and Better? Representativeness of the Influenza A Surveillance Using One Consolidated Clinical Microbiology Laboratory Data Set as Compared to the Belgian Sentinel Network of Laboratories
Source: Front Public Health. 2019 Jun 18;7:150. doi: 10.3389/fpubh.2019.00150 (PMC6591264; doi:10.3389/fpubh.2019.00150)

Supplemental Digital Content 2: Geographical representativeness of the LHUB-ULB data's in the flu infection's notification in the BSNL in 2016

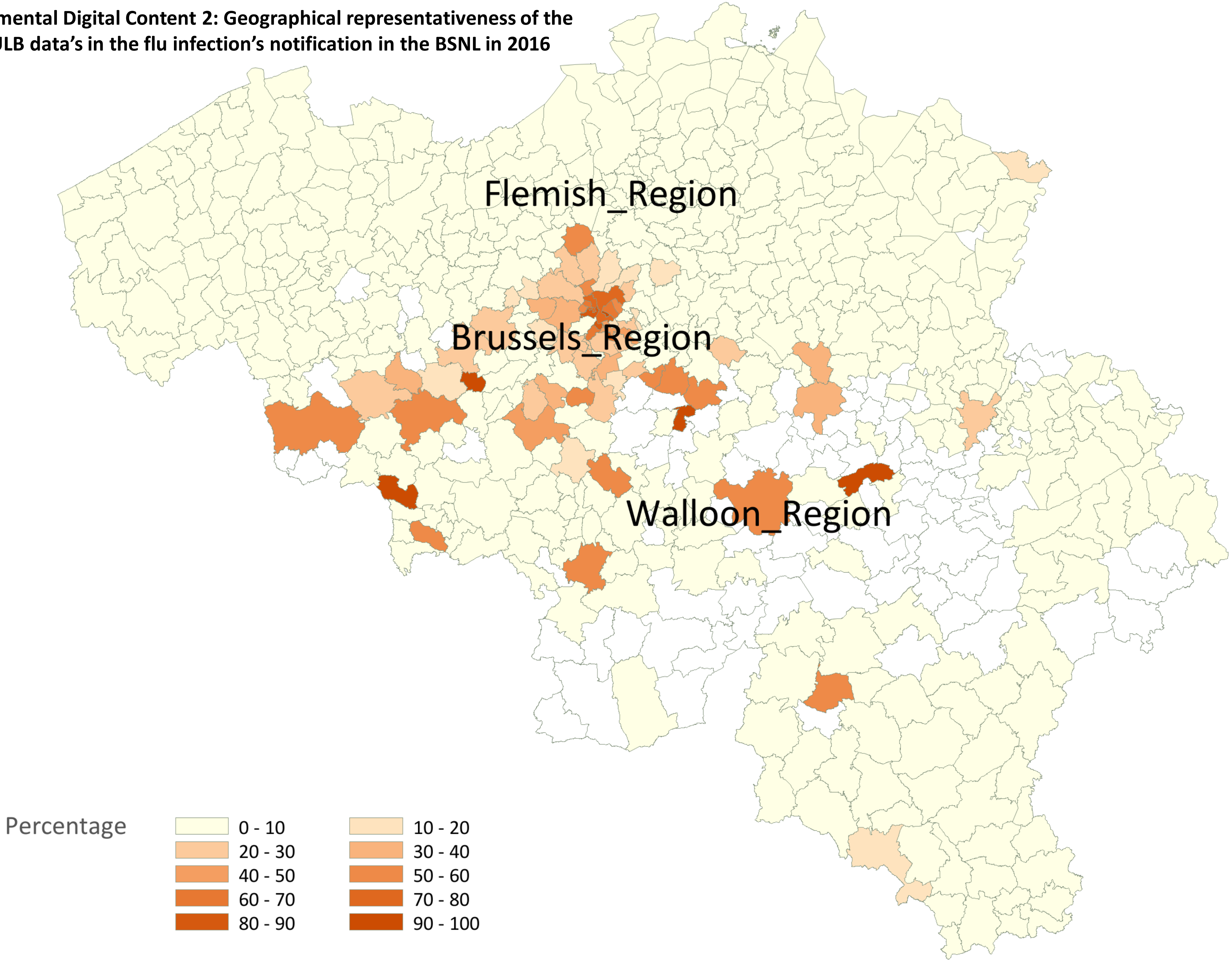

Supplement: Supplementary file 2 [file Data_Sheet_2.pdf]
