# Supplementary material for: Bigger and Better? Representativeness of the Influenza A Surveillance Using One Consolidated Clinical Microbiology Laboratory Data Set as Compared to the Belgian Sentinel Network of Laboratories
Source: Front Public Health. 2019 Jun 18;7:150. doi: 10.3389/fpubh.2019.00150 (PMC6591264; doi:10.3389/fpubh.2019.00150)
Supplement: Supplementary file 3 [file Data_Sheet_3.pdf]

Supplemental digital content 3:

Tableau 1 : Spearman's rank correlation between all the flu's cases notified by the LHUB-ULB and all the flu's cases notified by the BSNL, BSNL- and ILI consultation rates (national and per region) compared weekly during 2014-2017

	LHUB-ULB vs BSNL	LHUB-ULB vs BSNL-	LHUB-ULB vs ILI
**Belgium 2014-2017**	0.9114	0.8916	0.7816
**Brussels Region 2014-2017**	0.9668	0.9405	0.7180
**Flanders Region 2014-2017**	0.7776	0.7712	0.7255
**Walloon Region 2014-2017**	0.7139	0.7036	0.6301

Pvalue: < 0.0001 for all comparison $\rho_{Sp\ test}$

Tableau 2 : Spearman's rank correlation between all the flu's cases notified by the LHUB-ULB and all the flu's cases notified by the BSNL, BSNL- and ILI consultation rates (national and per region) compared weekly during the three influenza seasons.

	LHUB-ULB vs BSNL	LHUB-ULB vs BSNL-	LHUB-ULB vs ILI
**Belgium**			
**2014-2015 (w40/14 to w39/15)**	0.9041	0.8889	0.7580
**2015-2016 (w40/15 to w39/16)**	0.9151	0.8986	0.7956
**2016-2017 (w40/16 to w39/17)**	0.8613	0.8311	0.7779
**Brussels Region**			
**2014-2015 (w40/14 to w39/15)**	0.9697	0.9476	0.7224
**2015-2016(w40/15 to w39/16)**	0.9776	0.9545	0.6921
**2016-2017(w40/16 to w39/17)**	0.9293	0.8902	0.7637
**Flanders Region**			
**2014-2015(w40/14 to w39/15)**	0.8217	0.8171	0.7735
**2015-2016(w40/15 to w39/16)**	0.8874	0.8871	0.8294
**2016-2017(w40/16 to w39/17)**	0.6067	0.5877	0.5765
**Wallonia**			
**2014-2015 (w40/14 to w39/15)**	0.7263	0.7127	0.4972
**2015-2016 (w40/15 to w39/16)**	0.8428	0.8400	0.8347
**2016-2017 (w40/16 to w39/17)**	0.5785	0.5526	0.5801

Pvalue: < 0.0001 for all comparison $\rho_{Sp\ test}$
