# Supplementary material for: Bigger and Better? Representativeness of the Influenza A Surveillance Using One Consolidated Clinical Microbiology Laboratory Data Set as Compared to the Belgian Sentinel Network of Laboratories
Source: Front Public Health. 2019 Jun 18;7:150. doi: 10.3389/fpubh.2019.00150 (PMC6591264; doi:10.3389/fpubh.2019.00150)
Supplement: Supplementary file 4 [file Data_Sheet_4.pdf]

Supplemental digital content 4: Numbers and proportion of the different types and subtypes of Influenza analysed during three consecutive seasons (2014-2017) by the NIC in comparison with the LHUB-ULB

			NRC									LHUB-ULB								
				FLU A				FLU B					FLU A				FLU B			
			FLU	total	A H1	A H3	A NT	total	YAM	VIC	B NL	FLU	total	A H1	A H3	A NT	total	YAM	VIC	B NL
Season 2014-2015	from week 40/2014 (Sept 29th, 2014) to week 20/2015 (May 15th, 2015)	Number of positive samples	485	405	60	325	19	79	73	6	0	655	529	-	-	-	127	-	-	-
		Number of tested samples	932	485	405	405	405	485	79	79	0	5321	655	-	-	-	655	-	-	-
		Percentage (%)	52,0	83,5	14,8	80,2	4,7	16,3	92,4	7,6		12,3	80,8	-	-	-	19,4	-	-	-
Season 2015-2016	from week 40/2015 (Sept 28, 2015) to week 19/2016 (May 15, 2016)	Number of positive samples	381	186	173	2	11	197	6	190	1	880	427	-	-	-	465	-	-	-
		Number of tested samples	743	381	186	186	186	381	197	197	197	5580	880	-	-	-	880	-	-	-
		Percentage (%)	51,3	48,8	93,0	1,1	5,9	51,7	3,0	96,4	0,5	15,8	48,5	-	-	-	52,8	-	-	-
Season 2016-2017	from week 40/2016 (October 3rd, 2016) to week 12/2017 (March 26th, 2017)	Number of positive samples	331	331	3	322	6	1	1	0	0	606	597	-	-	-	9	-	-	-
		Number of tested samples	634	331	331	331	331	331	1	1	1	5840	606	-	-	-	606	-	-	-
		Percentage (%)	52,2	100,0	0,9	97,3	1,8	0,3	100,0	0,0	0,0	10,4	98,5	-	-	-	1,5	-	-	-

NT: not subtyped/ non subtypable - NL: no lineage determined
